# The association of payer type on genicular radiofrequency neurotomy treatment outcomes: Results of a cross-sectional study

**DOI:** 10.1016/j.inpm.2024.100407

**Published:** 2024-04-05

**Authors:** Samantha Braun, Jason Mascoe, Marc Caragea, Tyler Woodworth, Tim Curtis, Michael Blatt, Cole Cheney, Todd Brown, Daniel Carson, Keith Kuo, Dustin Randall, Emily Y. Huang, Andrea Carefoot, Masaru Teramoto, Amanda Cooper, Megan Mills, Taylor Burnham, Aaron Conger, Zachary L. McCormick

**Affiliations:** a Department of Physical Medicine and Rehabilitation, University of Texas San Antonio, San Antonio, TX, USA; b Department of Physical Medicine and Rehabilitation, University of Utah, Salt Lake City, UT, USA; c School of Medicine, University of Utah, Salt Lake City, UT, USA; d Department of Radiology, University of Utah, Salt Lake City, UT, USA; e Mayo Clinic Health System, Mankato, MN, USA; f Johns Hopkins University School of Medicine, Baltimore, MD, USA

**Keywords:** Knee pain, Genicular nerve, Radiofrequency ablation, Osteoarthritis, Payer type

## Abstract

**Background:**

Genicular radiofrequency neurotomy (GRFN) is an effective treatment for a subset of individuals with chronic knee pain. Previous studies demonstrate that Medicare and Medicaid beneficiaries report worse outcomes following various interventional procedures compared with commercially insured patients.

**Objective:**

Evaluate the association of payer type on GRFN treatment outcomes.

**Methods:**

Consecutive patients who underwent GRFN at a tertiary academic center were contacted for participation. Demographic, clinical, and procedural characteristics were collected from electronic medical records. Outcome data were collected by standardized telephone survey at 6–12 months, 12–24 months and ≥24 months. Treatment success was defined as ≥50% numerical pain rating scale (NPRS) score reduction from baseline. Data were analyzed using descriptive statistics for demographic, clinical, and procedural characteristics. Logistic and Poisson regression analyses were performed to examine the association of variables of interest and pain reduction.

**Results:**

One hundred thirty-four patients treated with GRFN (mean 65.6 ± 12.7 years of age, 59.7% female) with a mean follow-up time of 23.3 ± 11.3 months were included. Payer type composition was 48.5% commercial (n = 65), 45.5% Medicare (n = 61), 3.7% Medicaid (n = 5), 1.5% government (n = 2), and 0.8% self-pay (n = 1). Overall, 47.8% of patients (n = 64) reported ≥50% NPRS score reduction after GRFN. After adjusting for age, follow-up duration, Kellgren-Lawrence osteoarthritis grade, baseline opioid use, antidepressant/antianxiety medication use, history of knee replacement, and number of RFN lesions placed, the logistic regression model showed no statically significant association between payer type and treatment outcome (OR = 2.11; 95% CI = 0.87, 5.11; *p* = 0.098).

**Discussion/conclusion:**

In this study, after adjusting for demographic, clinical, and procedural characteristics, we found no association between payer type and treatment success following GRFN. This observation contrasts findings from other interventional studies reporting an association between payer category and treatment success.

## 1 Introduction

Osteoarthritis (OA) is the most common form of arthritis, involving degenerative changes to the protective cartilage and joint structures over time, leading to pain and functional impairments. The global prevalence of radiographically-confirmed symptomatic knee OA in 2010 was estimated to be 3.8% [1]. In 2008, nearly 27 million people were estimated to have clinically significant OA, which is one of the leading causes of disability in the US adult population [2]. OA has been shown to increase with age and additional risk factors such as obesity, specific occupations, previous injuries, and genetics [3]. It is anticipated that the prevalence of OA will increase globally as the population ages and obesity rates continue to rise [1].

Conservative treatment of painful knee OA includes exercise, self-management and education, weight loss, Tai Chi, bracing, physical therapy, topical and oral non-steroidal anti-inflammatory drugs (NSAIDS), and intra-articular (IA) corticosteroid injection [4]. In severe cases, total knee arthroplasty (TKA) is often performed. Risks associated with TKA include continued pain, infection, joint instability, periprosthetic fracture, and prosthetic joint failure [5]. Despite improvements in overall quality of life, approximately 20% of patients suffer from recalcitrant pain post-TKA [6].

Genicular radiofrequency neurotomy (GRFN) is a treatment for knee pain in patients who choose to avoid surgery, are not surgical candidates, or have post-TKA pain. GRFN was first studied in a prospective manner by Choi et al. [7]. The procedure involves partial sensory denervation of the anterior joint capsule through targeted delivery of radiofrequency energy to the genicular nerves [8]. Initially, GRFN protocols predominately focused on three nerves: the superior medial genicular nerve (SMGN) and inferior medial genicular nerve (IMGN), which innervate the medial aspect of the knee joint, along with the superior lateral genicular nerve (SLGN) that innervates the lateral aspect of the knee joint. However, recent anatomical studies have revealed a more extensive sensory innervation of the anterior knee joint capsule. Both Tran et al. and Fonkoue et al. identified 10 or more articular branches originating from the femoral, obturator, and sciatic nerves in independent dissection studies [9,10]. As more complete descriptions of knee joint neuroanatomy emerge, conventional GRFN protocols have been expanded to capture additional sensory targets and account for anatomic variation [11,12]. The majority of clinical outcome studies indicate that GRFN effectively reduces pain and improves function in patients with painful knee OA [[13], [14], [15]], with the most recent literature indicating that expanded GRFN protocols likely improve outcomes beyond that of the originally proposed technique [12,[16], [17], [18], [19]]. Although there are case reports of complications such as pes anserine tendon injury, septic arthritis, skin burns, and periarticular hematomas [[20], [21], [22], [23]], GRFN is generally regarded as a safe and well-tolerated procedure [24]. Positive outcomes have been associated with ≥80% pain relief with prognostic blocks [11,12,25], no opioid use, absence of coexisting mental health disorders, and lower baseline pain scores [12].

Patients in the United States can access various types of health insurance depending on their eligibility, including private (commercial) insurance and government-funded plans such as Medicaid and Medicare. There is a considerable body of literature investigating outcome discrepancies associated with payer type following various surgeries and interventional procedures. Badin et al. performed a systematic review of outcomes on elective spine surgery and found that Medicaid was associated with worse health outcomes, and increased emergency department utilization [26]. Additionally, a retrospective analysis of total hip arthroplasty (THA) by Shau et al. found Medicaid payer status was associated with increased resource usage and total cost following primary THA [27].

However, to our knowledge, the relationship between payer type and patient outcomes has not been evaluated for GRFN. As such, this study aimed to assess whether primary payer type was independently correlated with treatment success in patients who underwent GRFN for chronic knee pain.

## 2 Methods

### 2.1 Data collection

This study was conducted at a tertiary academic center. The protocol was approved by the University of Utah Institutional Review Board (IRB 00138414). Using CPT codes 64,624 and 64,640, electronic medical records (EMRs) of 226 consecutive patients who underwent GRFN from October 2015 to March 2021 were reviewed. Inclusion criteria were patients aged 18–80 years with painful knee OA or persistent post-TKA pain with at least one positive (≥50% pain relief) prognostic genicular nerve block prior to their GRFN procedure. Detailed descriptions of the genicular nerve block and GRFN procedural techniques were previously published [15]. Exclusion criteria included inability or refusal to participate in a phone call-based standardized outcome survey and missing or low (≤3 points) baseline Numeric Pain Rating Scale (NPRS) scores within two months before the index GRFN.

### 2.2 Procedures

Both prognostic genicular nerve blocks and GRFN procedures were performed as we have described previously [16].

#### 2.2.1 Data collection

Data collected at baseline from the EMR included age, body mass index, duration of pain, gender, smoking status, workers’ compensation status, active opioid prescription for ≥6 months at the time of GRFN, current use of antidepressants or anxiolytic medications, presence and laterality of knee replacement and number of nerves targeted by prognostic genicular blocks and the index GRFN procedure, radiofrequency probe type, prognostic block response, GRFN laterality, the number of prior GRFN procedures, and payer type. Payer type was based on the primary coverage listed in the EMR and included commercial (private) insurance, Medicare, Medicaid, other government plan, and self-pay.

Outcome data were collected at a single follow-up assessment conducted via telephone call using a standardized survey at variable time points categorized between 6 and 12 months, 12–24 months, or ≥24 months post-procedure, depending on when the phone call occurred relative to the GRFN procedure. The standardized telephone survey included NPRS scores at the time of follow up and self-reported improvement as measured by the Patient Global Impression of Change (PGIC) at the time of follow up for the most recent GRFN. Data was input into REDCap, which is a secure web-based platform designed for validated data capture in research studies [28,29]. The primary outcomes were the proportion of participants with ≥50% NPRS reduction and ≥ 2-point NPRS reduction. Secondary outcomes included the proportion of participants who reported a PGIC score of ≥6 (consistent with an improvement rating of at least “much improved”), NPRS follow-up scores, and opioid cessation. Core outcome measures and minimally important differences were chosen in accordance with established recommendations for studies involving measurement of chronic pain [[30], [31], [32]].

### 2.3 Data analysis

Patient demographics and clinical characteristics were summarized using descriptive statistics, including mean, standard deviation (SD), median, and interquartile range (IQR) for continuous variables, as well as frequency and percentage for categorical variables. A 95% confidence interval (CI) was also calculated for select variables. Contingency table analyses were used to examine whether responder rates differed by payer types for the following categorical outcome variables: (1) ≥ 50% NPRS reduction from baseline, (2) ≥ 2-point NPRS reduction from baseline, and (3) ≥ 6 on PGIC indicating “much improved” or “very much improved”. A one-sample *t*-test was used to determine if NPRS follow-up scores had improved significantly from baseline (*i.e.*, whether NPRS score reduction and percent change were significantly different from zero and 0%, respectively).

Multivariate logistic regression (LR) analysis was performed to explore the relationships between categorical outcome variables (≥50% NPRS reduction, ≥ 2-point NPRS reduction, and ≥6 on the PGIC) and payer type, while using select demographic and clinical characteristics as covariates. The included covariates were follow-up timeframe, worst compartment KL grade, opioid use at baseline, antidepressant/anxiolytic medication at baseline, history of knee replacement, number of nerves targeted by RFA, and age [11,12]. Odds ratios (ORs) and 95% CIs were calculated for each coefficient.

Lastly, a Poisson regression model was developed to explore the relationship between payer type and the continuous secondary outcome variable of NPRS score at follow-up. This model incorporated the same predictor variables as the LR models, with addition of baseline NPRS score as an extra covariate. Incidence rate ratios (IRRs) and 95% CIs were calculated for each coefficient. All analyses were conducted using Stata/MP 17.0 (StataCorp LLC, College Station, TX), with an *α* level of 0.05 for statistical significance.

## 3 Results

Of the 226 patients identified, a total of 134 participants were included in this study. Patient demographics and clinical characteristics, including payer types, are presented in Table 1a, Table 1ba and 1b. Commercial insurance (*n* = 65; 48.5%) and Medicare (*n* = 61; 45.5%) were the most common payer types. The remaining patients received primary coverage through Medicaid (*n* = 5; 3.7%) and other government insurance plans (*n* = 2; 1.5%), with one case (0.8%) of self-pay. For the majority of patients, follow-up occurred at 12–24 months (*n* = 60; 44.8%) and ≥24 months (*n* = 53; 39.5%) post-GRFN, whereas only 15.7% (*n* = 21) were contacted 6–12 months after the procedure. The average follow-up time was 23.4 ± 11.3 months.

**Table 1a:** Patient demographics and clinical characteristics (*N* = 134; continuous variables).

Continuous Variable	*N*	Mean (SD)	Min, Max
Age	134	65.6 (12.7)	34, 96
Height (cm)	107	168.9 (10.5)	147.3, 190.5
Weight (kg)	107	94.7 (26.3)	47.2, 161
BMI (kg/m^2^)	134	33.4 (7.9)	20.1, 59
Duration of pain (yr)	134	5.4 (5.8)	0.25, 49
Follow-up months	112	23.3 (11.3)	5, 56

Abbreviations: BMI = body mass index; Max = maximum value; Min = minimum value; SD = standard deviation.

**Table 1b:** Patient demographics and clinical characteristics (*N* = 134; categorical variables).

Categorical Variable	Frequency (%)
Payer type	
Commercial	65 (48.5)
Medicare	61 (45.5)
Medicaid	5 (3.7)
Other Government	2 (1.5)
Self-pay	1 (0.8)
Follow-up interval	
6–12 months	21 (15.7)
12–24 months	60 (44.8)
≥ 24 months	53 (39.5)
Gender	
Male	54 (40.3)
Female	80 (59.7)
Smoking	
Never	100 (74.6)
Current	3 (2.2)
Former	31 (23.1)
Opioid use at baseline	
Yes	37 (27.6)
No	97 (72.4)
Antidepressant/anxiolytic medication at time of GRFN	
Yes	63 (47.0)
No	71 (53.0)
Depression	
Yes	66 (49.3)
No	68 (50.7)
Anxiety	
Yes	55 (41.0)
No	79 (59.0)
Worker's compensation claim	
Yes	4 (3.0)
No	130 (97.0)
Worst compartment KL grade	
0	16 (11.9)
1	6 (4.5)
2	18 (13.4)
3	25 (18.7)
4	69 (51.5)
Prognostic block relief	
> 50%	14 (10.5)
50–79%	15 (11.2)
80–99%	23 (17.2)
100%	82 (61.1)
RFA probe type	
Cooled	129 (96.3)
Conventional monopolar	5 (3.7)
History of knee replacement in knee treated with GRFN	
Yes	25 (18.7)
No	109 (81.3)
Laterality of knee replacement	
Left	12 (9.0)
Right	13 (9.7)
Presence of lower extremity hardware	
Yes	9 (6.7)
No	125 (93.3)
GRFN laterality	
Left	74 (55.2)
Right	60 (44.8)
Number of previous GRFN procedures	
0	117 (87.3)
1	16 (11.9)
2	1 (0.7)
Number of nerves targeted with GRFN	
3	13 (9.7)
4	39 (29.1)
5	67 (50.0)
6	4 (3.0)
7	7 (5.2)
8	4 (3.0)

Abbreviations: GRFN = genicular radiofrequency neurotomy; KL = Kellgren and Lawrence; RFA = radiofrequency ablation.

Continuous NPRS scores at baseline and follow-up are summarized according to payer type in Table 2. The mean baseline NPRS score for all patients was 6.2 ± 1.7 (95% CI: 6.0, 6.5). NPRS score reductions at follow-up were significantly higher than zero (*p* < 0.05) for patients with commercial (2.5 ± 3.4 [95% CI: 1.6, 3.3]) and Medicare payer types (3.1 ± 3.7 [95% CI: 2.2, 4.0]), as the lower bounds of both 95% CIs were greater than zero. Likewise, percent NPRS reduction at follow-up was significantly greater than 0% (*p* < 0.05) for commercial and Medicare patients (36.4 ± 52.5% [95% CI: 23.5, 49.3] and 44.6 ± 54.4% [95% CI: 30.9, 58.4], respectively).

**Table 2:** Continuous NPRS scores at baseline and follow-up.

Outcome	Payer Type	*n*	Mean (SD)	Median (IQR)	Min, Max	95% CI (mean)
Baseline NPRS	Commercial	65	6.3 (1.5)	6 (5, 7)	4, 10	5.9, 6.7
	Medicare	61	6.1 (1.9)	6 (4, 7)	4, 10	5.6, 6.6
	Medicaid	5	6.6 (1.3)	6 (6, 8)	5, 8	5.4, 7.8
	Other Government	2	6.0 (1.4)	6 (5, 7)	5, 7	4.0, 8.0
	Self-pay	1	8.0 (*N/A*)	*N/A*		
	**Total**	134	6.2 (1.7)	6 (5, 8)	4, 10	**6.0, 6.5**
Follow-up NPRS	Commercial	65	3.9 (3.2)	4 (0, 7)	0, 10	3.1, 4.6
	Medicare	61	3.1 (2.9)	2 (0, 5)	0, 10	2.3, 3.8
	Medicaid	5	4.2 (1.9)	4 (3, 5)	2, 7	2.5, 5.9
	Other Government	2	3.0 (4.2)	3 (0, 6)	0, 6	−2.9, 8.9
	Self-pay	1	0.0 (*N/A*)	*N/A*		
	**Total**	134	3.5 (3.0)	3 (0, 6)	0, 10	**2.9, 4.0**
NPRS reduction (baseline NPRS minus follow-up NPRS)	Commercial*	65	2.5 (3.4)	2 (0, 5)	−5, 10	1.6, 3.3
	Medicare*	61	3.1 (3.7)	3 (0, 6)	−4, 10	2.2, 4.0
	Medicaid	5	2.4 (3.2)	2 (1, 5)	−2, 6	−0.4, 5.2
	Other Government	2	3.0 (5.7)	3 (−1, 7)	−1, 7	−4.9, 10.9
	Self-pay	1	8.0 (*N/A*)	*N/A*		
	**Total**	134	2.8 (3.5)	2 (0, 6)	−5, 10	**2.2, 3.4**
% NPRS change (from baseline to follow-up)	Commercial*	65	36.4 (52.5)	33 (0, 100)	−100, 100	23.5, 49.3
	Medicare*	61	44.6 (54.4)	50 (0, 100)	−100, 100	30.9, 58.4
	Medicaid	5	29.5 (45.2)	33 (16.7, 62.5)	−40, 75	−10.5, 69.5
	Other Government	2	40.0 (84.9)	40 (−20, 100)	−20, 100	−78.7, 158.7
	Self-pay	1	100.0 (*N/A*)	*N/A*		
	**Total**	134	40.4 (53.1)	40 (0, 100)	−100, 100	**31.3, 49.5**

* Significantly different from baseline according to one sample *t*-test (*p* < 0.05).

Abbreviations: CI = confidence interval; IQR = interquartile range; Max = maximum value; Min = minimum value; N/A = not applicable; NPRS = numeric pain rating scale; SD = standard deviation.

Responder rates for the three pre-defined categorical outcome variables are presented in Table 3a, Table 3b, Table 3c. Excluding the single patient with self-pay, NPRS responder rates were highest for the Medicare payer type group, with 57.4% (95% CI: 44.9, 69.0) and 63.9% (95% CI: 51.4, 74.8) of Medicare patients reporting ≥50% NPRS reduction and ≥ 2-point NPRS reduction, respectively. However, the 95% CIs for the proportion of responders in each group overlapped with one another, indicating that categorical improvements in NPRS pain scores did not differ significantly between payer types. Similarly, 60.0% of patients with commercial insurance (95% CI: 47.9, 71.0) and 55.7% of those with Medicaid (95% CI: 21.1, 88.2) reported a PGIC score of ≥6 points consistent with “improved” or “very much improved” at follow-up.

**Table 3a:** Proportion of participants with ≥50% NPRS reduction by payer type.

Payer type	≥50% NPRS reduction		95% CI (yes)
	Yes	No	
Commercial	25 (38.5)	40 (61.5)	27.6, 50.6
Medicare	35 (57.4)	26 (42.6)	44.9, 69.0
Medicaid	2 (40.0)	3 (60.0)	11.8, 76.9
Other Government	1 (50.0)	1 (50.0)	9.5, 90.5
Self-pay	1 (100.0)	0 (0.0)	20.7, 100.0
**Total**	**64 (47.8)**	**70 (52.2)**	**39.5, 56.2**

Note: values are frequency (%).

Abbreviations: CI = confidence interval; NPRS = numeric pain rating scale.

**Table 3b:** Proportion of participants with ≥2-point NPRS reduction by payer type.

Payer type	≥2-point NPRS reduction		95% CI (yes)
	Yes	No	
Commercial	38 (58.5)	27 (41.5)	46.3, 69.6
Medicare	39 (63.9)	22 (36.1)	51.4, 74.8
Medicaid	3 (60.0)	2 (40.0)	23.1, 88.2
Other Government	1 (50.0)	1 (50.0)	9.5, 90.5
Self-pay	1 (100.0)	0 (0.0)	20.7, 100.0
**Total**	**82 (61.2)**	**52 (38.8)**	**52.7, 69.0**

Note: values are frequency (%).

Abbreviations: CI = confidence interval; NPRS = numeric pain rating scale.

**Table 3c:** Proportion of participants with ≥6 on PGIC by payer type.

Payer type	≥6 on PGIC		95% CI (yes)
	Yes	No	
Commercial	39 (60.0)	26 (40.0)	47.9, 71.0
Medicare	34 (55.7)	27 (44.3)	43.3, 67.5
Medicaid	3 (60.0)	2 (40.0)	23.1, 88.2
Other Government	2 (100.0)	0 (0.0)	34.2, 100.0
Self-pay	1 (100.0)	0 (0.0)	20.7, 100.0
**Total**	**79 (59.0)**	**55 (41.0)**	**50.5, 66.9**

Note: values are frequency (%).

Abbreviations: CI = confidence interval; PGIC = Patient Global Impression of Change (scores ≥6 indicate at least “much improved”).

According to results from the logistic regression (LR) analyses, payer type was not significantly associated with ≥50% NPRS reduction, ≥ 2-point NPRS reduction, or ≥ 6 on PGIC after adjusting for covariates (*p* > 0.05; Table 4a). However, two of the LR models contained statistically significant covariates: a follow-up time of ≥24 months was a significant predictor variable for ≥50% NPRS reduction (OR = 3.36 [95% CI: 1.04, 10.85]; *p* = 0.042), while a history of knee replacement in the knee treated with GRFN was associated with lower odds by 68% of achieving a PGIC score ≥6 points (OR = 0.32 [95% CI: 0.12, 0.88]; *p* = 0.027).

**Table 4a:** Logistic regression models on ≥ 50% NPRS reduction, ≥ 2-point NPRS reduction, and ≥6 PGIC.

Outcome	Predictor	OR	95% CI	*p*
≥50% NPRS reduction^a^	Payer type (vs. commercial)			
	Medicare	2.11	0.87, 5.11	0.098
	Medicaid	1.42	0.20, 9.90	0.726
	Other Government	2.86	0.14, 56.75	0.490
	Self-pay*	–	–	–
	Follow-up (vs. 6–12 months)			
	12–24 months	2.11	0.68, 6.53	0.197
	≥24 months	3.36	1.04, 10.85	**0.042**
	Worst compartment KL grade (vs. 0–1)			
	2-3	3.50	0.97, 12.61	0.055
	4	3.12	0.89, 10.94	0.076
	Opioid use at baseline (vs. no)			
	Yes	0.64	0.27, 1.52	0.316
	Antidepressant/anxiolytic medication at time of GRFN (vs. no)			
	Yes	0.69	0.31, 1.51	0.354
	History of knee replacement in knee treated with GRFN (vs. no)			
	Yes	0.59	0.2, 1.74	0.342
	Number of nerves targeted with GRFN (vs. 3)			
	>3	1.20	0.31, 4.68	0.796
	Age	1.02	0.98, 1.05	0.376
≥2-point NPRS reduction^b^	Payer type (vs. commercial)			
	Medicare	1.40	0.59, 3.34	0.450
	Medicaid	1.26	0.19, 8.51	0.814
	Other Government	0.57	0.03, 11.00	0.708
	Self-pay*	–	–	–
	Follow-up (vs. 6–12 months)			
	12–24 months	1.37	0.48, 3.96	0.558
	≥24 months	2.17	0.71, 6.63	0.172
	Worst compartment KL grade (vs. 0–1)			
	2-3	1.69	0.54, 5.25	0.365
	4	1.85	0.61, 5.62	0.278
	Opioid use at baseline (vs. no)			
	Yes	0.84	0.37, 1.95	0.693
	Antidepressant/anxiolytic medication at time of GRFN (vs. no)			
	Yes	0.51	0.24, 1.11	0.092
	History of knee replacement in knee treated with GRFN (vs. no)			
	Yes	0.74	0.28, 1.98	0.548
	Number of nerves targeted with GRFN (vs. 3)			
	>3	2.04	0.57, 7.29	0.272
	Age	0.99	0.96, 1.02	0.573
≥6 PGIC^c^	Payer type (vs. commercial)			
	Medicare	0.80	0.34, 1.89	0.613
	Medicaid	1.09	0.14, 8.60	0.934
	Other Government*	–	–	–
	Self-pay*	–	–	–
	Follow-up (vs. 6–12 months)			
	12–24 months	0.92	0.31, 2.77	0.886
	≥24 months	0.55	0.18, 1.69	0.300
	Worst compartment KL grade (vs. 0–1)			
	2-3	0.62	0.18, 2.16	0.457
	4	0.51	0.15, 1.72	0.276
	Opioid use at baseline (vs. no)			
	Yes	1.04	0.45, 2.40	0.919
	Antidepressant/anxiolytic medication at time of GRFN (vs. no)			
	Yes	0.88	0.40, 1.91	0.742
	History of knee replacement in knee treated with GRFN (vs. no)			
	Yes	0.32	0.12, 0.88	**0.027**
	Number of nerves targeted with GRFN (vs. 3)			
	>3	3.23	0.85, 12.33	0.087
	Age	1.00	0.97, 1.04	0.962

Abbreviations: CI = confidence interval; OR = odds ratio; GRFN = genicular radiofrequency neurotomy; KL = Kellgren and Lawrence; NPRS = numeric pain rating scale; PGIC = Patient Global Impression of Change (scores ≥6 indicate at least “much improved”).

* Unable to reliably estimate regression coefficients due to insufficient number of cases.

As with the LR analyses, payer type was not a significant predictor variable in the Poisson regression model for NPRS follow-up score after accounting for the selected covariates, which included baseline NPRS score (*p* > 0.05; Table 4b). Compared to the LR models, the Poisson regression model contained a larger number of statistically significant predictor variables (*p* < 0.05), including worst compartment KL grade, baseline opioid use, and antidepressant/antianxiety medication at the time of GRFN. Patients with a worst compartment KL grade of 2–3 and 4 (vs. 0–1) were expected to have lower NPRS scores at follow-up by 32% (IRR = 0.68 [95% CI: 0.52, 0.88]; *p* = 0.004) and 35% (IRR = 0.65 [95% CI: 0.50, 0.84]; *p* = 0.001), respectively, after controlling for the other covariates. Conversely, baseline opioid use and antidepressant/anxiolytic medication at the time of GRFN were significantly associated with higher follow-up NPRS scores by 42% (IRR = 1.42 [95% CI: 1.17, 1.73]; *p* = 0.001) and 33% (IRR = 1.33 [95% CI: 1.09, 1.62]; *p* = 0.005), respectively. Although a follow-up time of ≥24 months (IRR = 0.75 [95% CI: 0.57, 0.99]) and >3 nerves (vs. 3) targeted by RFA (IRR = 0.75 [95% CI: 0.57, 0.99]) demonstrated statistical significance (*p* < 0.05), the 95% CIs of the IRRs were fairly close to 1.00, indicating that the effects of these predictor variables on follow-up NPRS score were likely minimal.

**Table 4b:** Poisson regression model on continuous NPRS score at follow-up.

Outcome	Predictor	IRR	95% CI	*p*
Follow-up NPRS	Baseline NPRS	0.95	0.90, 1.01	0.122
	Payer type (vs. commercial)			
	Medicare	0.89	0.71, 1.11	0.301
	Medicaid	0.99	0.63, 1.55	0.958
	Other Government	0.70	0.31, 1.62	0.411
	Self-pay*	<0.001	–	0.982
	Follow-up (vs. 6–12 months)			
	12–24 months	0.86	0.66, 1.12	0.264
	≥24 months	0.75	0.57, 0.99	**0.039**
	Worst compartment KL grade (vs. 0–1)			
	2-3	0.68	0.52, 0.88	**0.004**
	4	0.65	0.50, 0.84	**0.001**
	Opioid use at baseline (vs. no)			
	Yes	1.42	1.17, 1.73	**0.001**
	Antidepressant/anxiolytic medication at time of GRFN (vs. no)			
	Yes	1.33	1.09, 1.62	**0.005**
	History of knee replacement in knee treated with GRFN (vs. no)			
	Yes	1.06	0.83, 1.34	0.658
	Number of nerves targeted with GRFN (vs. 3)			
	>3	0.75	0.56, 0.99	**0.042**
	Age	0.99	0.98, 1.00	0.094

Abbreviations: CI = confidence interval; GRFN = genicular radiofrequency neurotomy; IRR = incidence rate ratio; KL = Kellgren and Lawrence; NPRS = numeric pain rating scale.

* 95% CI could not be calculated due to insufficient number of observations (only one case of self-pay).

## 4 Discussion

This study evaluated whether patient reported outcomes following treatment with GRFN varied by payer type. Initial analysis revealed that commercially-insured and Medicare patients experienced significantly greater reductions in NPRS scores compared to other payer types. However, after adjusting for demographic, clinical, and procedural characteristics, there was no significant association between payer type and treatment success, which was defined as NPRS score reductions of either ≥50% or ≥2 points from baseline. This finding was unexpected, as a large body of existing literature reports that Medicare and Medicaid beneficiaries have worse outcomes following various surgeries and interventional procedures compared to patients with commercial insurance.

Outcomes after interventional treatments for musculoskeletal pain may be influenced by payer type. Reisinger et al. evaluated ODI scores of 606 patients who underwent lumbosacral transforaminal epidural steroid injection for radicular pain [33]. Medicare and Medicaid payer types were found to be negative predictors of functional improvement after adjusting for other demographic, clinical, and procedural variables. Compared to those with commercial insurance, Medicare and Medicaid patients were significantly less likely to attain the minimal clinically important difference (MCID) of ≥15-point and ≥30% improvements in ODI scores.

When assessing outcomes of total knee replacement, Veltre et al. found that Medicare patients were at higher risk of mortality and post operative complications, including wound dehiscence, as compared to privately insured patients [34]. Similar outcomes were reported by Tirumala et al. when assessing major and minor complications following revisions of both total hip and knee joint arthroplasty [35]. In addition to higher cost burden and longer hospital stay, Medicaid beneficiary status was linked to a higher prevalence of wound dehiscence, hematoma, and postoperative in-hospital infection compared to non-Medicaid payer types [36].

Given the aging demographic of the United States and the anticipated increase in the prevalence of knee OA, GRFN presents itself as a compelling and viable treatment option for managing pain associated with knee OA. In a randomized clinical trial, Davis et al. found that, compared to IA corticosteroids, patients treated with cooled RFN experienced significantly greater pain reduction at six months as well as improvements in Oxford Knee Score (OKS), Global Perceived Effect, and analgesic use [13]. In a retrospective analysis of 31 patients who underwent GRFN, the procedure was shown on average to provide greater than 60% pain relief in patients for as long as 6 months [14]. In a systematic review, Fogarty et al. reported that 49–74% of participants achieved ≥50% pain relief at 6 months after undergoing GRFN treatment, which outperformed IA injections of steroid and hyaluronic acid, respectively [15]. Approximately half of patients in the present study reported a ≥50% reduction in NPRS score at an average follow-up time of 23.4 ± 11.3 months post-GRFN, suggesting that patients who undergo GRFN in “real-world” practice can be expected to report a similar rate of treatment success to that observed in clinical trials, particularly when expanded lesioning protocols are utilized. Importantly, the present observations were derived from a “real-world” patient cohort that included characteristics such as prior TKA, opioid usage, and significant depression, which were among the exclusion criteria for several clinical trials that have served to establish the effectiveness of GRFN [7,13,19].

We previously described negative and positive prognostic factors, as well as additional covariates associated with GRFN treatment success, for this study cohort in a separate publication [16].

Several important limitations to this study must be addressed. Of the 226 charts selected for review, only 134 patients completed follow-up telephone questionnaires and were thus included in this cross-sectional study. We found a higher probability of significant pain relief in those who were contacted after two years. It is possible that those who reported pain reduction at that distant time point might have had other treatment or those who did not benefit from the index procedure were lost to follow up. This sample size is smaller compared to similar studies evaluating the relationship between payer type and clinical outcomes across hundreds of thousands of patients and large studies will ultimately be required to confirm the present findings [34,36]. As a result of the reduced sample size and decreased power, potential smaller difference in outcomes based on payer status may have been overlooked. The generalizability of our results is also limited by the fact that all study participants were patients treated at a single academic medical center in Utah and future multicenter studies may be warranted. Additionally, Medicaid payer status (n = 5) was markedly underrepresented in our study population; only 5 participants reported it as their primary payer type compared to 65 patients with commercial insurance and 61 with Medicare. While the study did not include variables like household income and highest education level, it is worth considering that the socioeconomic diversity in this cohort might not have been extensive. This consideration is based on the fact that as of 2022, Utah had the lowest income inequality ranking among all 50 United States [37]. However, the precise extent of this influence remains uncertain. It would be prudent to conduct further research to ascertain further whether GRFN treatment outcomes differ according to payer type in more diverse populations, where a wider range of social and economic backgrounds are represented.

## 5 Conclusion

We present the results of a cross-sectional study evaluating whether GRFN treatment outcomes vary by patient payer type. In contrast to previous literature reports, this study found no significant associations between payer type and patient reported outcomes after adjusting for demographic, clinical, and procedural characteristics. Overall, GRFN was found to be an effective pain-relieving modality for symptomatic knee OA or persistent post-TKA pain. Further investigations, especially prospective studies collecting outcome data from large, diverse patient populations, are warranted to extend the clinical applications of the present findings.

## Conflict of interest statement

Zachary L. McCormick, MD serves on the Board of Directors of the International Pain and Spine Intervention Society and has received research grants from Avanos Medical (paid directly to the 10.13039/100007747University of Utah). Taylor Burnham, DO, is a consultant for Avanos Medical and has received grant funding from DIROS Technology (paid directly to the 10.13039/100007747University of Utah). Aaron Conger, DO, has received research grants from Stratus Medical (paid directly to the 10.13039/100007747University of Utah). There are no other potential conflicts of interest to disclose on the part of any of the other authors.

## Funding

This study was funded by research grants from (1) the Skaggs Foundation for Research and (2) the Mitchell and June Morris Foundation.

## Declaration of competing interest

The authors declare the following financial interests/personal relationships which may be considered as potential competing interests:

Zachary McCormick reports financial support was provided by Skaggs Foundation for Research. Zachary McCormick reports financial support was provided by Mitchell and June Morris Foundation. Zachary McCormick reports a relationship with International Pain and Spine Intervention Society that includes: board membership. Zachary McCormick reports a relationship with Avanos Medical Inc that includes: funding grants. Taylor Burnham reports a relationship with Avanos Medical Inc that includes: consulting or advisory. Taylor Burnham reports a relationship with Diros Technology Inc that includes: funding grants. Aaron Conger reports a relationship with Stratus that includes: funding grants. If there are other authors, they declare that they have no known competing financial interests or personal relationships that could have appeared to influence the work reported in this paper.
